# Web alert: Metagenomic mining for biotechnology

**DOI:** 10.1111/1751-7915.14334

**Published:** 2023-08-22

**Authors:** Lawrence P. Wackett

**Affiliations:** ^1^ Department of Biochemistry, Molecular Biology & Biophysics, BioTechnology Institute University of Minnesota St. Paul Minnesota USA

Mining terpene synthases

https://biotechnologyforbiofuels.biomedcentral.com/articles/10.1186/s13068‐022‐02189‐9

Terpenes are an industrially important and highly abundant class of natural products. This review discussed mining of soil microbial genomes for genes encoding known and novel terpenoid compounds.

Metagenomic analysis for new pectinases

https://biotechnologyforbiofuels.biomedcentral.com/articles/10.1186/s13068‐017‐0885‐y

This study looked a specific microbiome connected to apple compost to find new pectinolytic enzymes.

Repurposing CRISPRi for metagenomic mining

https://www.nature.com/articles/s41587‐022‐01563‐0

This study in *Nature Biotechnology* describes a new CRISPR‐interference method to rapidly mine metagenomic and genomic sequences. This journal is not available without subscription, but is available through many institutional libraries.

Metagenomic mining for natural products

https://www.biorxiv.org/content/10.1101/2021.01.20.427441v1.full

This article on a preprint server for biological studies describes a tool for rapid mining of microbial biosynthetic gene clusters.

Mining enzyme diversity

https://link.springer.com/referenceworkentry/10.1007/978‐3‐319‐31421‐1_216‐1

This review book chapter focuses on metagenomic mining for new enzyme diversity.

Microfluidics and metagenome mining

https://www.liebertpub.com/doi/10.1089/crispr.2022.0054

This paper describes a high‐throughput, microfluidic method to enrich rare sequences in a metagenomic mixture.

Mining hidden biosynthetic potential

https://www.nature.com/articles/s41467‐021‐24133‐5

This is a review article in an open‐source journal, *Nature Communications,* focused on mining for secondary metabolites.

Long‐read metagenomics of marine microbes

https://journals.asm.org/doi/10.1128/spectrum.01501‐23

Long‐read sequencing is important for discovering biosynthetic gene clusters, which are often comprised of dozens of contiguous genes.

Hunting down enzymes

https://elifesciences.org/digests/70021/hunting‐down‐enzymes

This magazine style article describes tools for analysing genomes to find new enzymes that modify DNA.

MGnify database

https://www.ebi.ac.uk/metagenomics

MGnify is a database and platform that can be used to assemble, analyse and archive microbiome‐derived nucleic acid sequences.

MGnify genomes

https://www.sciencedirect.com/science/article/pii/S0022283623000724

This recent paper describes MGnify Genomes, an extension of the MGnify database. This resource includes the increasing availability of biome‐specific metagenome‐assembled genomes (MAGs).

Metagenome submission guide

https://www.ncbi.nlm.nih.gov/genbank/metagenome/

This page offers information for submitting metagenome project sequences to the National Center for Biotechnology Information (NCBI).

